# SPATIAL PROFILING CELLULAR SENESCENCE IN THE HUMAN BRAIN ACROSS EIGHT DECADES OF LIFE

**DOI:** 10.1093/geroni/igad104.0731

**Published:** 2023-12-21

**Authors:** Miranda Orr

**Affiliations:** Wake Forest University School of Medicine, Winston-Salem, North Carolina, United States

## Abstract

Neurons with tau-containing neurofibrillary tangles (NFTs) closely correlate with Alzheimer’s disease (AD) progression and severity, but do not undergo immediate cell death. Our prior work determined that NFT-bearing neurons undergo a change in cell fate consistent with cellular senescence. CDKN2D/p19 was identified as a key biomarker for identifying senescent neurons in postmortem human AD brain tissue. Studying the interaction between senescent (e.g., p19+/NFT+) neurons and their environments is a necessary next step to understanding how these cells may be impacting their local and synaptically-connected neighboring cells. The goal of this project was to evaluate the expression and subcellular localization of multiple analytes in normal and senescent cells in postmortem mouse and human brains using non-destructive, multi-plex, spatially-resolved profiling methods. Postmortem mouse and human brains were analyzed using NanoString, Inc GeoMx digital spatial profiling (DSP) and CosMx spatial molecular imaging (SMI). Region of interest (ROI) selection included the presence or absence of neuronally expressed AT8 (phosphorylated tau, NFTs) and/or p19 (senescence). NFTs with or without p19 expression were compared to healthy neurons from the same cases. The expression of 86 proteins associated with neuropathology, inflammation, autophagy and cell typing was determined for each ROI and immediate environment using GeoMx. CosMx SMI was used to develop a subcellular expression map of >1000 genes and spatially-resolved cell type map of mouse and human entorhinal cortex and hippocampus. The complete dataset consisted of over 160,000 single cells and ~170 million transcripts, and a spatially-resolved cell type map of mouse brain tissue. Significant differences across biological aging pathways between disease and healthy conditions in both species were identified. Additionally, the results provide insights into which cells are susceptible to senolytic clearing interventions currently underway (NCT04685590).

